# Mitotic Catastrophe Occurs in the Absence of Apoptosis in p53-Null Cells with a Defective G1 Checkpoint

**DOI:** 10.1371/journal.pone.0022946

**Published:** 2011-08-10

**Authors:** Michalis Fragkos, Peter Beard

**Affiliations:** Ecole Polytechnique Fédérale de Lausanne, Swiss Institute for Experimental Cancer Research, Lausanne, Switzerland; University of Kansas Medical Center, United States of America

## Abstract

Cell death occurring during mitosis, or mitotic catastrophe, often takes place in conjunction with apoptosis, but the conditions in which mitotic catastrophe may exhibit features of programmed cell death are still unclear. In the work presented here, we studied mitotic cell death by making use of a UV-inactivated parvovirus (adeno-associated virus; AAV) that has been shown to induce a DNA damage response and subsequent death of p53-defective cells in mitosis, without affecting the integrity of the host genome. Osteosarcoma cells (U2OSp53DD) that are deficient in p53 and lack the G1 cell cycle checkpoint respond to AAV infection through a transient G2 arrest. We found that the infected U2OSp53DD cells died through mitotic catastrophe with no signs of chromosome condensation or DNA fragmentation. Moreover, cell death was independent of caspases, apoptosis-inducing factor (AIF), autophagy and necroptosis. These findings were confirmed by time-lapse microscopy of cellular morphology following AAV infection. The assays used readily revealed apoptosis in other cell types when it was indeed occurring. Taken together the results indicate that in the absence of the G1 checkpoint, mitotic catastrophe occurs in these p53-null cells predominantly as a result of mechanical disruption induced by centrosome overduplication, and not as a consequence of a suicide signal.

## Introduction

Apoptosis is a crucial mechanism in eliminating cells with unrepaired DNA damage and preventing carcinogenesis. It is characterized by a p53-dependent induction of pro-apoptotic proteins, leading to permeabilization of the outer mitochondrial membrane, release of apoptogenic factors into the cytoplasm, activation of caspases and subsequent cleavage of various cellular proteins. Apoptogenic effects include chromatin condensation and exposure of phosphatidylserine on membrane surfaces of the cell [1].

Caspases have a major role in executing apoptosis. They are synthesized as inactive proenzymes that become activated by cleavage [2]. Caspase-3 is the most important protease in the caspase-dependent apoptosis pathway, as it is required for chromatin condensation and fragmentation [3], [4]. Poly-ADP ribose polymerase (PARP-1) is a major target of caspase-3, since cleavage-mediated inactivation of PARP-1 preserves cellular ATP that is required for apoptosis [4], [5]. Although caspases constitute a substantial component of the apoptotic pathway, there is evidence that a caspase-independent apoptosis pathway also exists [6]. This pathway involves the apoptosis-inducing factor (AIF), which translocates from the mitochondria to the nucleus to cause chromatin condensation [7], [8], [9].

Cell death can also occur in the absence of apoptosis, by alternative non-apoptotic killing pathways, including autophagy and necrosis [10], [11]. Autophagy is a lysosomal mechanism of self-digesting cytosolic components, characterized by the conversion of the protein marker LC3A to LC3B [12], [13]. Non-apoptotically mediated death is occasionally named necrosis, to indicate cell death that is uncontrolled [14]. When necrosis occurs under regulation, it is called necroptosis [15]. Necrosis is mediated by DNA degradation, membrane distortion and cellular swelling [11].

Cell death in mitosis, also known as mitotic catastrophe, occurs as a consequence of failure to complete mitosis. In that case, cells proceed into mitosis after a transient cell cycle arrest and fail to separate, leading to catastrophic cell division [16]. Catastrophic mitosis can also take place as a result of centrosome overduplication and consequent entry into mitosis with multiple spindle poles [17]. The G2 checkpoint is crucial for preventing mitotic cell death and when it is aborted, mitotic catastrophe is potentiated [11], [18]. Mitotic catastrophe is often characterized by the formation of giant micronucleated cells, which reflects the abnormal segregation of chromosomes.

Although there are a number of studies linking apoptosis to mitotic catastrophe in one way or another, the relationship between mitotic catastrophe and apoptosis remains unclear. Several studies have shown that mitotic cell death involves activation of caspases, cytochrome c release, chromatin condensation and DNA degradation [16], [19]–[22]. On the other hand, other studies have concluded that death in mitosis is an apoptosis-independent event that may be followed independently by apoptosis [23], [24]. A recent review of mitotic catastrophe concluded that there is no broad consensus on the use of this term, and that mitotic catastrophe can lead either to an apoptotic morphology or to necrosis [25].

In this work we have probed cell death in mitosis by using a virus, adeno-associated virus (AAV), which is a replication-defective parvovirus containing a 4.7 kb single-stranded DNA genome [26]. AAV can induce a DNA damage response in the host cell that is attributable to the inability of the virus to complete its replication [27]. The DNA damage signaling pathway, induced by stalled replication forks on the viral replication origins, has been shown to lead to significant cell death in mitosis in different types of p53-deficient cancer cells [28], [29]. This effect of the virus has been demonstrated not to be caused by the viral protein products, since UV-inactivated AAV leads to a stronger DNA damage response than the untreated virus [30].

It has been suggested that p53-independent mechanisms of killing tumor cells may not involve apoptosis and could be a result of induced mechanical damage, rather than programmed cell death [31]. The aim of the study reported here was to investigate whether cell death in mitosis requires apoptosis or can be a consequence of mechanical collapse of cells undergoing aberrant mitosis. To induce mitotic cell death, we used UV-inactivated AAV, an agent that activates a cellular DNA damage signaling pathway without causing DNA damage on the host genome [32]. Infection of p53-deficient osteosarcoma U2OS cells (U2OSp53DD cells, which contain a dominant-negative p53 mutation [30] led to significant levels of mitotic cell death that we show to be caspase-independent. Infected U2OSp53DD cells did not show signs of chromatin condensation or DNA fragmentation and were negative for other apoptotic markers. Consistent with these findings, time-lapse microscopy studies indicated cell death due to mechanical damage. In comparison, as a control for the apoptotic markers, MO59K glioblastoma cells tested in parallel responded to the infection by entering a cell death program with all the hallmarks of apoptosis.

## Results

### AAV-infected p53-deficient U2OS cell death is associated with aberrant mitosis

**Figure 1:**
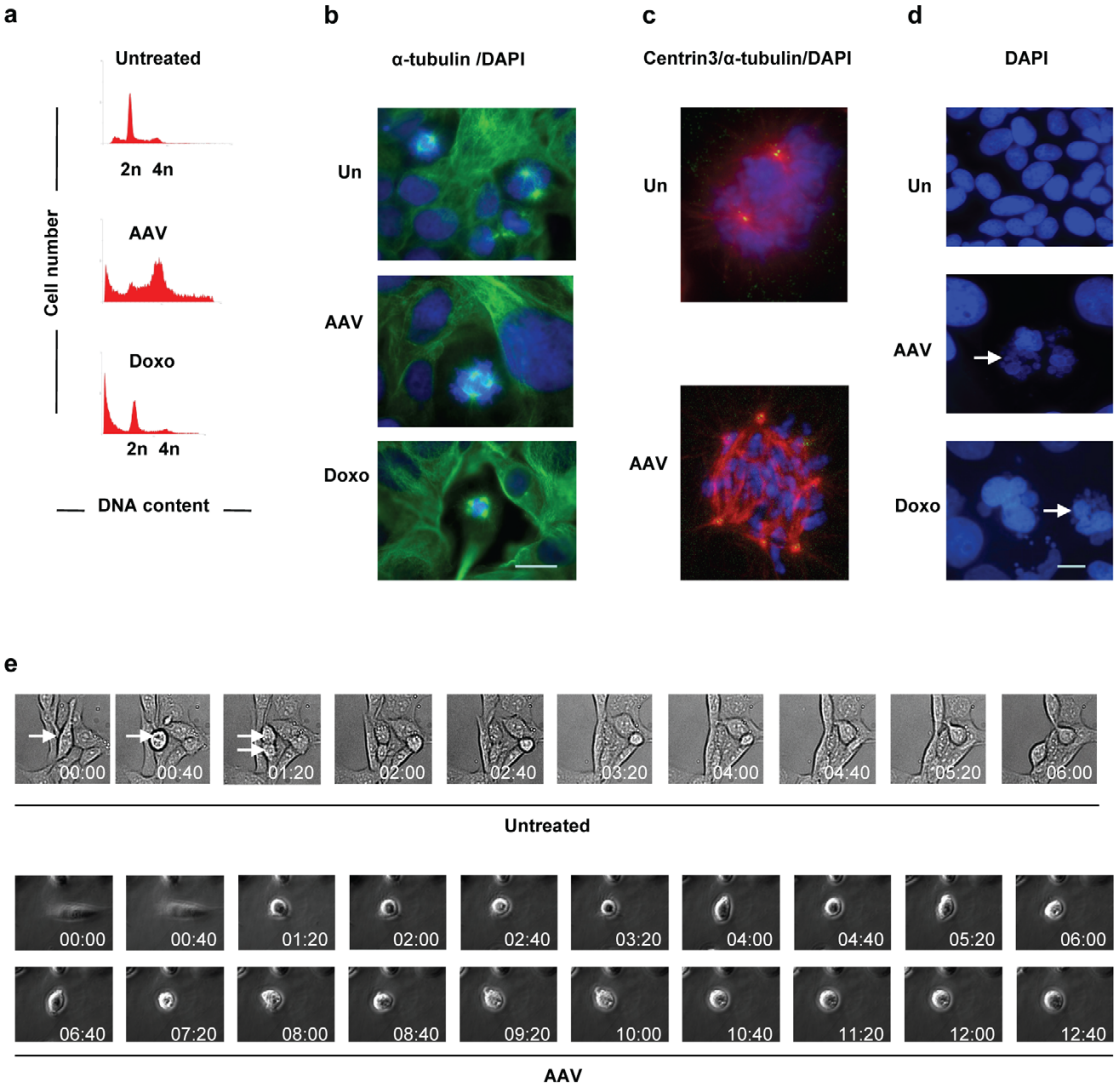
U2OSp53DD cells infected with AAV die by mitotic catastrophe. (a) U2OSp53DD cells die 4 days after infection. Cells were infected with AAV and analyzed by PI staining and FACS 4 days post-infection (x-axis: DNA content; y-axis: cell count). Doxorubicin (Doxo) leads to cell death in mitosis and was used as a control. (b) AAV-infected cells show multiple spindle poles. U2OSp53DD cells were infected and then stained for α-tubulin 4 days post-infection. Bar: 30 µm. (c) Infected and control uninfected cells were stained for DNA with DAPI, for α-tubulin (red) and for centrin3 (green). Merged red and green give yellow. (d) U2OSp53DD cells were infected with AAV and then stained with DAPI 4 days post-infection. The arrows highlight micronucleated cells. Bar: 15 µm. (e) Prolonged mitosis can lead to cell death. Cells were infected with AAV and analyzed by time-lapse brightfield-microscopy 2 days post-infection. Images were acquired using the 20× objective and phase-contrast was used for the infected cells. The arrows indicate a normally-dividing cell.

We have previously shown that infection of U2OS cells with UV-inactivated AAV leads to cell cycle arrest at G2 [30]. Cells that lack the p53 pathway however respond differently to the virus. When U2OSp53DD cells were infected, then stained with propidium iodide (PI) and analyzed by fluorescence-activated cell sorting (FACS), the infection was seen to cause cell death; dead cells are indicated by the presence of a subG1 cell population [28], [30]. In Figure 1a, 37% of the cells were in the subG1 region following the AAV infection, while this proportion was 6% in the uninfected control. To further confirm and characterize this cell death, infected cells were analyzed by immuno-fluorescence (IF) and time-lapse microscopy.

Infected U2OSp53DD cells were stained for α-tubulin, which is a component of microtubules and therefore of the mitotic spindle poles. The cells were counterstained with DAPI and analyzed by IF microscopy. The images obtained showed frequent multipolar mitoses, indicative of abnormal mitosis (Figure 1b) and very clear at higher magnification (Figure 1c). These results were confirmed by experiments showing that over 70% of the infected cells contained multiple (>2) centrosomes, and that 58% of infected cell mitoses showed evidence of multipolar spindles, both values compared to less than 10% in uninfected controls [29]. The overall mitotic index of attached cells changes relatively little (less than 2-fold) following AAV infection [28], [29]. Interestingly, the response of the cells to the virus was similar to that induced by the topoisomerase-II inhibitor doxorubicin, which has been reported to induce mitotic cell death [33], although less accumulation of G2 cells was seen, possibly because in this case the analysis was done 4 days post-treatment and the G2-arrested cells died.

**Figure 2:**
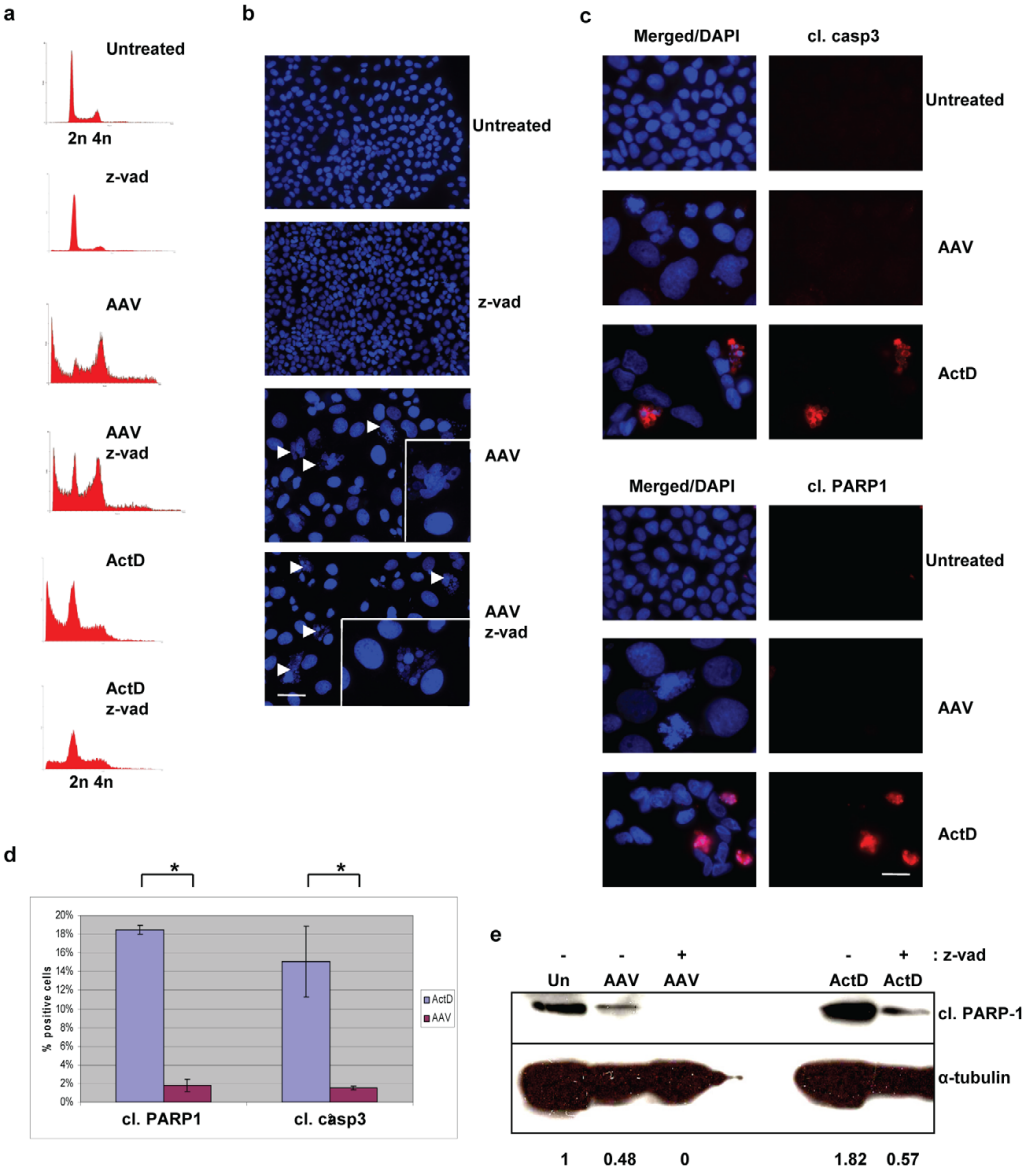
Mitotic cell death induced by AAV is caspase-independent. (a) FACS analysis showing that inhibition of caspases does not prevent cell death. U2OSp53DD cells were infected with AAV and then treated with zVAD-fmk 2 days post-infection. The cells were analyzed by PI staining and FACS, 4 days post-infection (x-axis: DNA content; y-axis: cell count). Treatment with zVAD-fmk prevented the ActD-induced cell death that was used as a control. Treatment with zVAD-fmk alone did not have an effect on the cell cycle. (b) Caspase inhibition does not prevent micronucleation. AAV-infected U2OSp53DD cells were treated with zVAD-fmk 2 days post-infection and analyzed by DAPI staining and IF 4 days after infection. Arrows indicate micronucleated cells. Images were acquired using a 10× objective. Bar: 215 µm. The inserts show cells at higher magnification. (c) AAV-infected U2OSp53DD cells are negative for cleaved caspase-3 (casp3) and cleaved PARP-1. Cells were infected and stained for the two markers of caspase-dependent apoptosis and DAPI, 4 days after infection. Bar: 25 µm. (d) The percentage of cleaved caspase-3 and cleaved PARP-1 –positive cells from the experiment described in (c). Error bars represent standard deviations from two independent experiments. The asterisk denotes statistically significant difference (2-tailed t-test) (e) Western blotting showing low levels of cleaved PARP-1 in AAV-infected U2OSp53DD cells. Treatment with zVAD-fmk decreased the levels of cleaved PARP-1. α-tubulin was used as a loading control. The relative levels of cleaved PARP-1 normalized using the loading controls are shown below. Un: untreated.

Staining of attached infected U2OSp53DD cells with 4,6-diamidino-2-phenylindole (DAPI) also revealed significant numbers of micro-nucleated cells (Figure 1d); in a series of experiments the average number of micro-nucleated cells ranged from 8% to 14% (see also Figures 2 and 5), whereas they were not seen in controls. The way the cells die was examined by time-lapse microscopy. U2OSp53DD cells were infected and analyzed under a light microscope, with images acquired every 5 min during 16 h. Live-imaging showed that the infected cells are blocked in mitosis for a prolonged period (12.5 h on average). During this time they appear to be repeatedly distorted by internal forces, unsuccessfully try to divide and finally die without detectably exiting mitosis (Figure 1e and the time-lapse images in Movie S1). In untreated cells, in contrast, mitosis was completed in about 1.5 h (Movie S2). We therefore conclude from the results of FACS, IF and time-lapse microscopy experiments that death of the AAV-infected p53-deficient U2OS cells is for the most part associated with aberrant mitosis.

### AAV-induced mitotic cell death is caspase-independent

To investigate whether mitotic cell death induced by AAV involves apoptosis, U2OSp53DD cells were infected with AAV and treated with the pan-caspase inhibitor carbobenzoxy-vanyl-ala-nyl-aspartyl-[O-methyl]-fluoromethyl-ketone (zVAD-fmk). Cells were then stained with PI and analyzed by flow cytometry (Figure 2a). In the AAV-infected sample 48% of the cells were in the subG1 population, while with uninfected cells this population was almost undetectable. In the zVAD-fmk-treated AAV-infected sample the subG1 population was still prominent, comprising 34% of the cells. Thus inhibition of caspases did not prevent cell death, as indicated by the continued presence of the subG1 population in the zVAD-fmk-treated AAV-infected cells. However, the proportion of subG1 cells was reduced following zVAD-fmk treatment. We therefore conclude that the major part of AAV-induced mitotic cell death is caspase-independent, although a minority of cells may die by a caspase-dependent pathway, presumably apoptotic. This duality will be discussed below. In actinomycin D (ActD) -treated cells, on the other hand, zVAD-fmk essentially abolished cell death. ActD, by inhibiting transcription, acts as a potent inducer of caspase-dependent apoptosis and was used as a positive control for apoptosis. Infected U2OSp53DD cells treated with zVAD-fmk were also analyzed by DAPI staining and IF to identify micronucleated cells (Figure 2b). This experiment confirmed that caspase inhibition does not prevent the appearance of micronucleated cells, since the zVAD-fmk-treated AAV-infected sample showed a comparable number of micronucleated cells (11%) to that of the AAV-infected one (8%). It is also apparent in Figure 2b that the AAV-infected nuclei are vastly enlarged compared to uninfected controls. This is a very reproducible finding though the enlargement factor is variable, presumably depending on time, cell-type or MOI. Cell size can thus be a useful marker of AAV infection.

The involvement of caspases in the AAV-induced death of U2OSp53DD cells was further examined by staining infected cells for cleaved caspase-3 and cleaved poly-ADP-ribose polymerase (PARP) -1, which are markers of caspase-dependent apoptosis (Figure 2c). AAV-infected cells did not show condensed chromatin or stain positively for cleaved caspase-3 and cleaved PARP-1. On the other hand, a significant number of the control ActD-treated apoptotic cells did have condensed and fragmented chromatin and, at the same time, were positive for both apoptotic markers. The numbers of cleaved caspase-3 and cleaved PARP-1–positive cells were counted in the AAV –infected and ActD-treated samples and the percentage of positive cells in each sample was calculated. As shown in Figure 2d, only a small population of AAV-infected cells showed signs of caspase-dependent apoptosis (2%), whereas 18% (as indicated by cleaved PARP-1) and 15% (as indicated by cleaved caspase-3) of ActD-treated cells were dying of caspase-dependent apoptosis. The levels of cleaved PARP-1 were also examined in AAV-infected and ActD-treated U2OSp53DD cells by total protein extraction and western blotting (Figure 2e). Cleaved PARP-1 levels did not rise after infection with AAV, whereas they increased significantly after ActD-mediated apoptosis. Treatment with zVAD-fmk diminished the amount of cleaved PARP-1 in both cases. Taken together, these data indicate that the way the U2OSp53DD cells die after infection with AAV is largely independent of caspases, although we do not exclude that a minority of cells may die in a caspase-dependent manner.

### AAV-induced mitotic cell death is independent of apoptosis and autophagy

**Figure 3:**
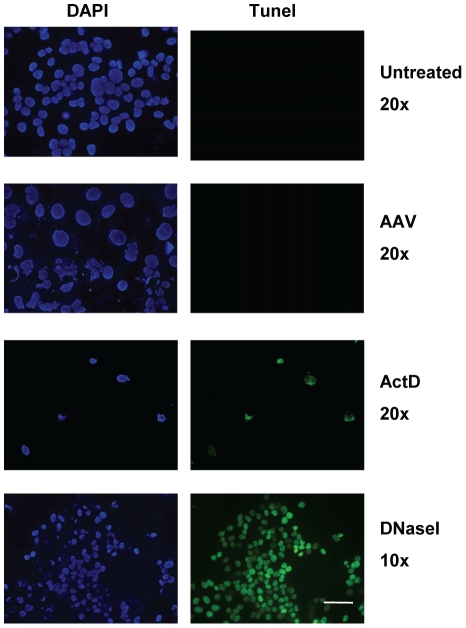
AAV-infected U2OSp53DD cells show no signs of DNA fragmentation. Tunel assay showing absence of DNA fragmentation in U2OSp53DD cells infected with AAV. Cell nuclei were counterstained with DAPI. ActD was used as a control for apoptotic DNA breaks. Cells were analyzed 4 days after infection or ActD-treatment. Cells were also treated with DNaseI for 30 min after fixation and used as controls. Bar: 75 µm (20× images) or 150 µm (10× images).

**Figure 4:**
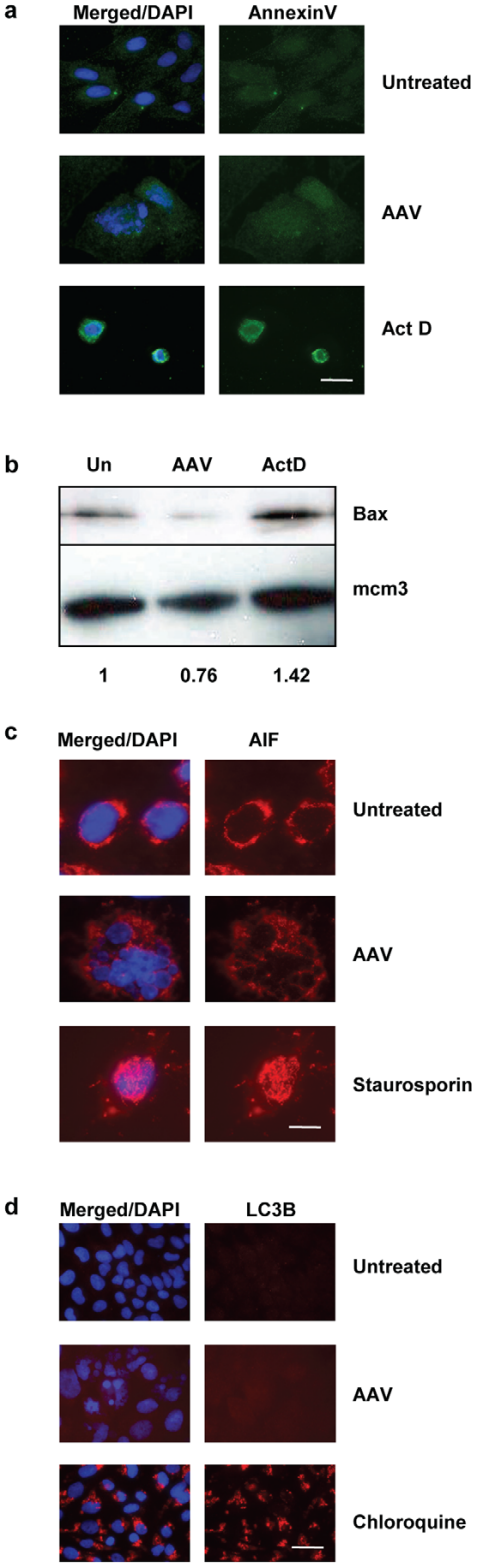
AAV-induced cell death is independent of apoptosis and autophagy. (a) IF analysis of apoptotic cells (4 days post-infection) using an annexin-V-fluorescein conjugate. The AAV-infected U2OSp53DD cells are negative for this staining, unlike the ActD-treated cells that were used as positive controls. DNA was stained with DAPI. Bar: 30 µm. (b) AAV-infection in U2OSp53DD cells does not increase the levels of Bax. Total protein was extracted from infected cells 4 days post-infection and examined for Bax levels by western blotting. Mcm3 was used as a loading control. Un: untreated. Quantification of the Bax signal relative to the loading control is shown below. (c) AAV-infected U2OSp53DD cells are negative for AIF. Cells were infected and analyzed by IF 4 days post-infection. DAPI was used to stain the nuclei. Staurosporin-treated cells were used as a positive control. Bar: 85 µm. (d) U2OSp53DD cells infected with AAV are negative for the autophagy marker LC3B. Nuclei were visualized by DAPI-staining. Cells were analyzed by IF analysis 4 days after infection. Chloroquine was used as a positive control. Bar: 35 µm.

The next step in our study was to investigate whether AAV-infected U2OSp53DD cells show signs of chromosome fragmentation that would imply apoptosis. For this purpose, a tunel assay was performed and the results (Figure 3) demonstrated that AAV-infected cells do not incorporate DNA breaks, unlike the positive control ActD- treated cells, which were tunel-positive. Consistent with the tunel result, genomic DNA extracted from the AAV-infected cells was not seen to be degraded on gel electrophoresis (data not shown). To investigate further whether apoptosis is involved in the mitotic cell death induced by AAV, infected U2OSp53DD cells were stained for membrane-exposed phosphatidylserine, using annexin-V conjugated to fluorescein. Cells infected with AAV and analyzed for fluorescence showed no staining for annexin-V, while the positive controls showed clear staining, indicating that AAV-infected U2OSp53DD cells die in an apoptosis-independent manner (Figure 4a). Absence of apoptotic signaling was further confirmed in AAV-infected cells by assaying for the levels of a pro-apoptotic protein marker, Bax (Figure 4b). Quantification showed that AAV-infection did not lead to elevated levels of Bax that would come as a result of apoptosis. On the other hand, treatment with ActD resulted in an increase in Bax levels. Finally, to examine if caspase-independent apoptosis is activated in U2OSp53DD cells after infection with AAV, infected cells were analyzed for nuclear localization of the apoptosis-inducing factor (AIF) by IF (Figure 4c). Nuclear staining of AIF was only observed in control staurosporin-treated cells but in none of the AAV-infected cells.

In order to investigate whether autophagy is induced after infection of U2OSp53DD cells with AAV, infected cells were stained for the protein marker LC3B, which is produced only when autophagy is initiated. The infected cells, analyzed by IF microscopy, were negative for LC3B, indicating that they do not die of autophagy (Figure 4d). On the other hand, cells treated with chloroquine, a known inducer of autophagy, exhibited elevated levels of LC3B. These data thus suggest that the way the U2OSp53DD cells die after AAV infection is independent of autophagy.

### AAV-infected cells do not die by necroptosis

**Figure 5:**
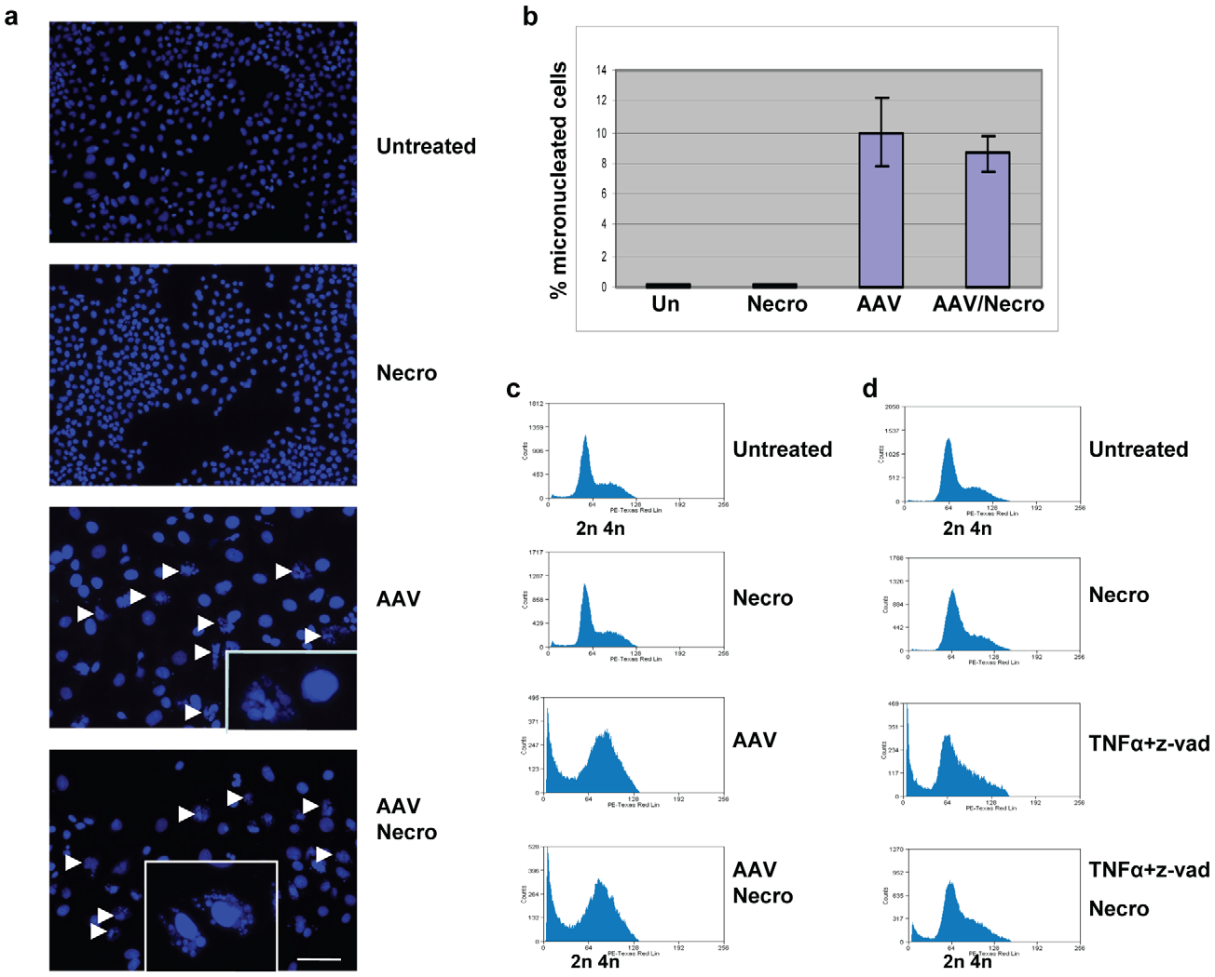
AAV-induced cell death is independent of necroptosis. (a) DAPI staining showing micronucleated cells among infected U2OSp53DD cells treated with necrostatin-1 (necro). Necrostatin-1 treatment alone did not have a significant effect on the viability of the cells. Images were acquired using the 10× objective. Arrows indicate micronucleated cells. The inserts show cells at a higher magnification. Bar: 230 µm. (b) The experiment described in (a) was replicated and the average percentage of micronucleated cells was calculated in infected cells with or without necrostatin-1 treatment. Error bars represent standard deviations from two independent experiments. Un: untreated. (c) PI staining and FACS analysis showing that AAV-induced cell death in U2OSp53DD cells is not sensitive to necrostatin-1 (x-axis: DNA content; y-axis: cell count). Necrostatin-1 treatment alone did not have an effect on the cell cycle. (d) Control FACS experiment to test the effectiveness of necrostatin-1. NIH3T3 cells were treated with TNFα and zVAD-fmk to induce necroptosis, which was then successfully inhibited by necrostatin-1. These experiments were performed with the DAKO flow cytometer (see Materials and Methods) and so have a slightly different presentation from the other FACS analyses.

We then asked the question whether the U2OSp53DD cells die by programmed necrosis, also known as necroptosis. To investigate this hypothesis we used a potent inhibitor of necroptosis, called necrostatin-1 [15]. Cells were infected and, at the same time, treated with necrostatin-1. DAPI staining and microscopic analysis were performed 4 days post-infection, and showed that the formation of large numbers of micronucleated cells still occurred despite the necrostatin-1 treatment (Figure 5a). The experiment was replicated and the average percentage of micronucleated cells was calculated (Figure 5b). The effect of necrostatin-1 on the viability of the infected cells was then examined by cell cycle analysis using flow cytometry. Cells were infected with AAV with or without necrostatin-1 and analyzed 4 days post-infection by PI staining and FACS. As shown in Figure 5c, necrostatin-1 did not affect the cell death-inducing potential of AAV, indicated by the continued presence of the subG1 peak in addition to the broad G2 peak, demonstrating that the U2OSp53DD cells do not die of necroptosis after infection with AAV. To control for the effectiveness of necrostatin-1, NIH3T3 cells were treated with tumor necrosis factor alpha (TNFα) and zVAD-fmk, so as to specifically induce necroptosis. The cells were then treated, or not, with necrostatin-1, to inhibit necroptosis, and were analyzed 3 days post-treatment by flow cytometry (Figure 5d). Necrostatin-1 indeed inhibited cell death induced by TNFα and zVAD-fmk. Taken together, these data clearly suggest that, by the criterion of sensitivity to necrostatin-1, U2OSp53DD cell death after AAV infection does not depend on necroptosis.

The absence of apoptosis, autophagy and necroptosis in AAV-infected U2OSp53DD cells led us to consider the hypothesis that these cells collapse during cell division, due to crucial mechanical problems caused by the presence of the overduplicated centrosomes. To examine this, U2OSp53DD cells were infected and treated with nocodazole, to prevent microtubule polymerization and so to relax the pulling forces that we considered may contribute to the mitotic death in these cells. The results (Figure S1) showed that the number of micronucleated cells decreased significantly in AAV-infected cells after nocodazole treatment. We therefore conclude that relaxation of the microtubule pulling forces during mitosis can suppress at least the role of micronucleation in infected U2OSp53DD cell death.

### Mitotic catastrophe or apoptosis following AAV infection is cell-dependent

**Figure 6:**
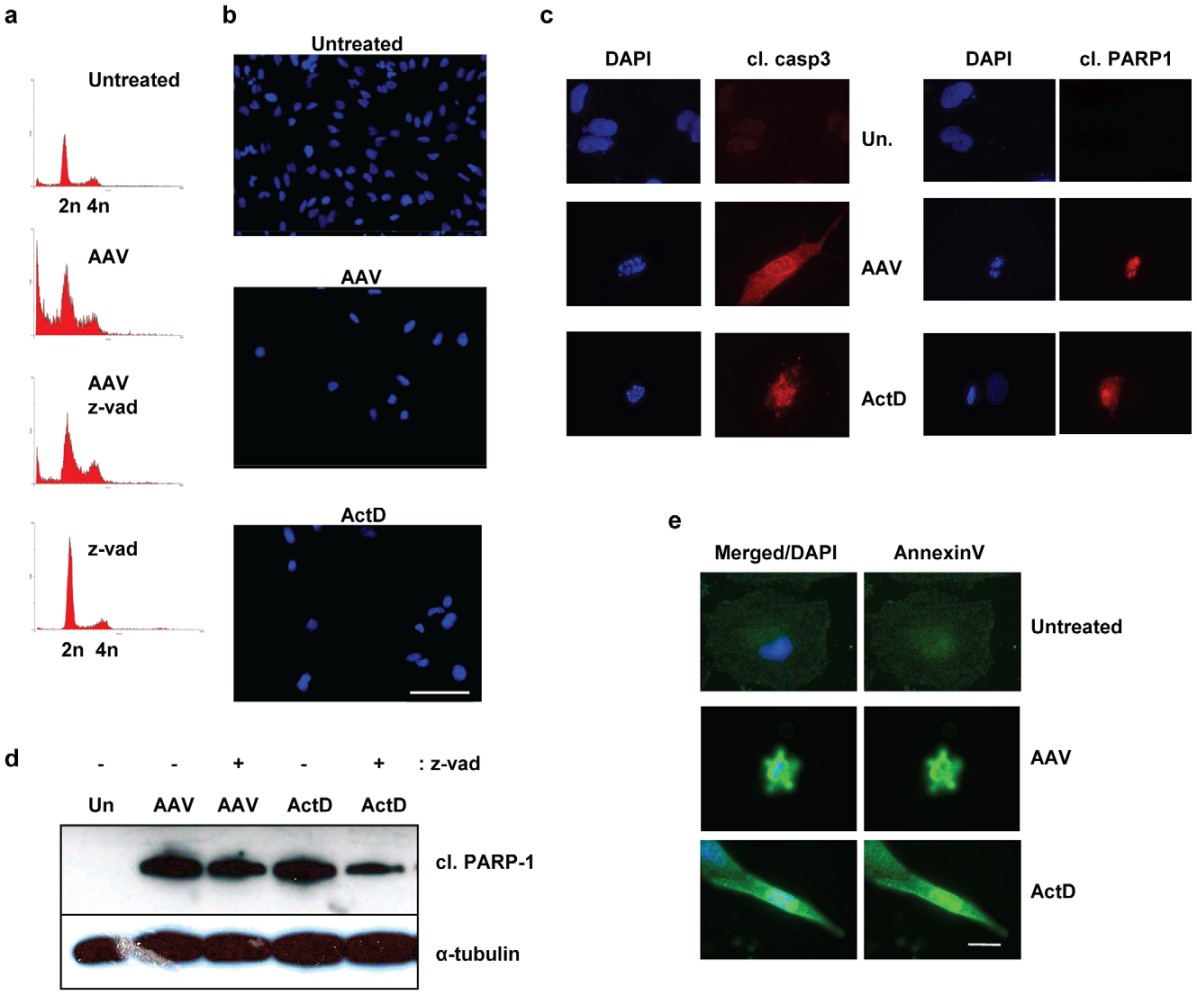
AAV induces apoptosis in M059K cells. (a) Inhibition of caspases leads to a decrease in the SubG1 population induced by AAV. M059K cells were infected, treated with zVAD-fmk and then stained with PI and analyzed by flow cytometry 4 days after infection (x-axis: DNA content; y-axis: cell count). Treatment with zVAD-fmk alone did not have a significant effect on the cells. (b) Infection with AAV does not lead to micronucleation in M059K cells. Infected cells were stained with DAPI 4 days post-infection. ActD was used as a control with no micronucleation and positive for apoptosis. Bar: 115 µm. (c) AAV-infected M059K cells have condensed or fragmented chromatin and are positive for cleaved caspase-3 and cleaved PARP-1. Cells were infected and analyzed by IF 4 days post-infection. DAPI was used to stain the nuclei. Bar: 35 µm. (d) Western analysis showing that glioblastoma cells are positive for cleaved PARP-1 after infection with AAV. Protein levels were assayed 4 days after infection. α-tubulin was used as a loading control. (e) AAV-infected M059K cells are positive for annexin-V staining. Cells were analyzed by IF 4 days post-infection, as in Figure 4. DNA was stained with DAPI. Bar: 30 µm.

Can these conclusions of mitotic cell death following AAV infection be generalized to other cancer cell types? Are the tests we have used to detect apoptosis after AAV infection reliable? To answer these questions, different cancer cell types have been examined for their response to AAV. We have shown that p53-deficient colon cancer cells (HCT116 p53−/−), but not the homologous cells containing p53, also die predominantly in mitosis, similarly to U2OSp53DD [28], thus demonstrating that our conclusions are not limited to this cell-type. Infected Saos-2 cells, on the other hand, can undergo rapid apoptosis following infection [30]. We therefore tested, in parallel with the experiments on U2OSpp53DD cells, the effects of AAV infection on the glioblastoma cells, MO59K. These cells were found to respond to AAV infection by displaying all the hallmarks of apoptosis, so establishing the validity of the apoptosis assays we used. Thus, staining with PI followed by FACS analysis showed that M059K cells die after infection with AAV, as indicated by the presence of a subG1 DNA peak (44% of cells, Figure 6a). Inhibition of caspases by treatment with zVAD-fmk markedly reduced the killing capacity of the virus, as indicated by the diminished subG1 population (8% of cells) in the AAV-infected zVAD-fmk-treated samples. To further investigate how the glioblastoma cells are killed by AAV, infected cells were stained with DAPI and examined by microscopy 4 days later (Figure 6b). IF analysis did not show a significant number of micro-nucleated cells in the AAV-infected sample, confirming that these cells differ from U2OSp53DD cells in their response to AAV infection.

Further examination of the infected M059K samples revealed cells with condensed chromatin, consistent with these cells dying of apoptosis. To confirm this, M059K cells were infected and stained for the presence of cleaved caspase-3 and cleaved PARP-1. IF analyses (Figure 6c) showed that many of the infected and ActD-treated cells, but none of the control untreated cells, were positive for these markers. The levels of PARP-1 were further studied by extracting total protein and performing a western blot (Figure 6d). Infected M059K cells showed elevated levels of cleaved PARP-1, similar to those of ActD-treated cells. Treatment with zVAD-fmk diminished the levels of cleaved PARP-1, slightly in the case of AAV and to a greater extent with ActD. Finally, M059K cells were stained for annexin-V-fluorescein binding and analyzed by IF microscopy. The infected cells were positive for this apoptotic marker, as were the ActD-treated cells. Therefore, taken together these data clearly indicate that the glioblastoma cells die 4 days after infection by undergoing caspase-dependent apoptosis.

**Figure 7:**
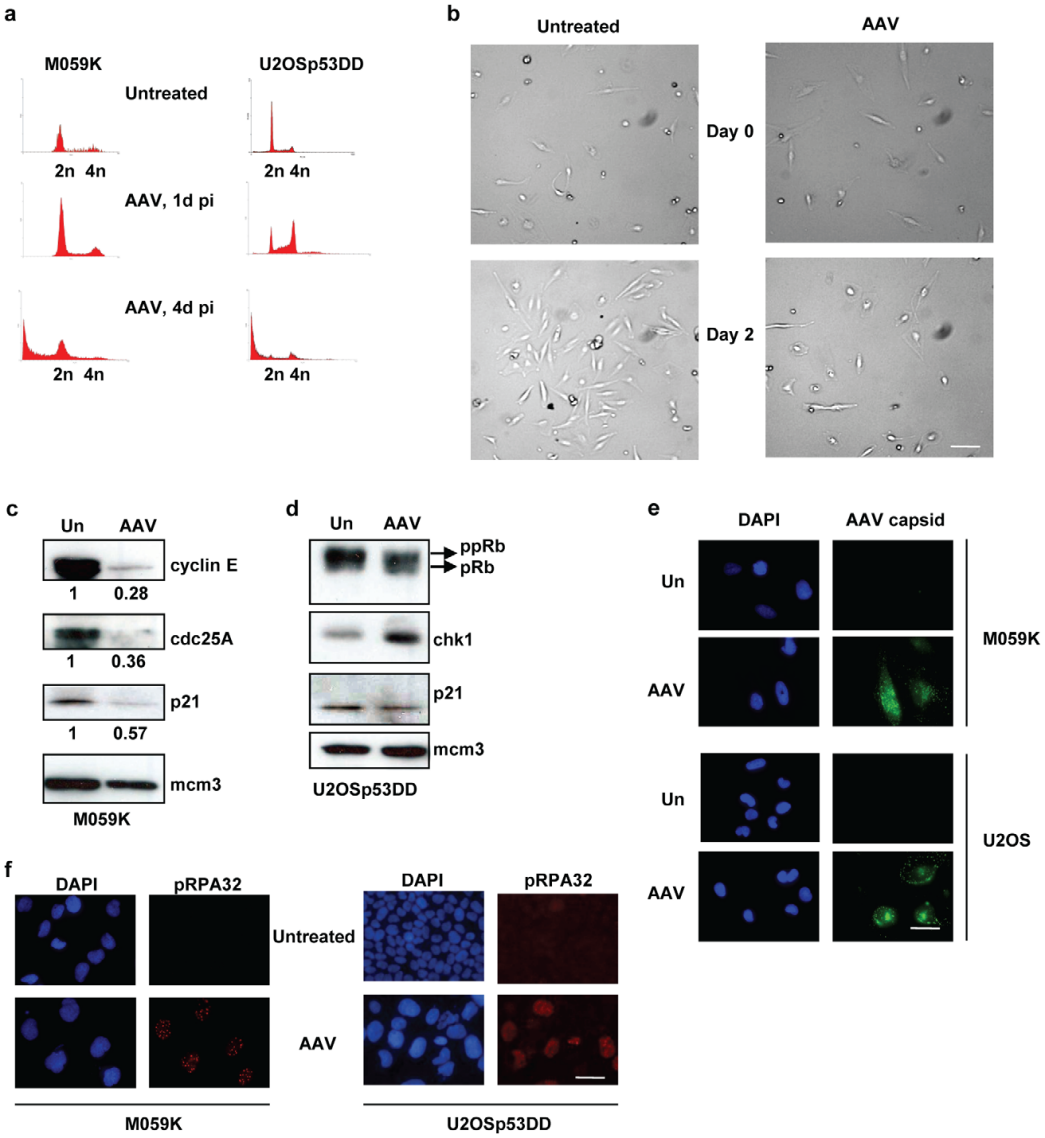
M059K cells have, unlike U2OSp53DD cells, a functional G1 checkpoint. (a) PI staining and FACS analysis of AAV-infected M059K and U2OSp53DD cells (x-axis: DNA content; y-axis: cell count). U2OSp53DD cells arrest at G2 1 day post-infection, whereas M059K cells do not. Both cell types proceed to death 4 days after infection. (b) Brightfield-microscopy images of untreated and AAV-infected M059K cells showing that infected cells arrest up to 2 days after infection. Images were acquired using the 10× objective. Bar: 230 µm. (c) M059K cells arrest in G1 after AAV-infection. Cells were infected and total protein was extracted 1 day post-infection. Western blotting showed low levels of cyclin E and cdc25A in infected cells, which is indicative of a functional G1 checkpoint. Mcm3 was used as a loading control. The numerical values below the bands are the relative protein amounts normalized to the loading control. Un: untreated. (d) The same experiment was performed in U2OSp53DD cells. High levels of phosphorylated pRb (ppRb) and of chk1 1 day post-infection indicate that these cells have a weak G1 checkpoint and arrest in G2. (e) Control experiment showing that M059K cells are efficiently infected with AAV. Cells were infected with untreated-AAV and stained using an AAV-capsid antibody, 1 day after infection. U2OSp53DD cells were used as a control. DAPI was used to stain nuclei. Bar: 35 µm. (f) Control IF experiment showing that AAV induces a similar DNA damage response in M059K cells to that induced in U2OSp53DD cells. Induction of phospo-RPA32 (pRPA32) foci was used as a marker for DNA damage response. DNA was stained using DAPI. The experiment was performed 1 day post-infection. Bar: 35 µm.

In order to look further into the different behavior of glioblastoma and osteosarcoma cells in response to AAV, we examined the cell cycle checkpoints in these two types of cell. Cells from both lines were infected and then analyzed by PI staining and FACS analysis, 1 day post-infection. M059K cells showed a G1 peak, whereas U2OSp53DD cells arrested strongly at G2 (Figure 7a). Both cell types underwent cell death 4 days post-infection. To test whether the G1 peak of M059K infected cells corresponded to a G1 arrest, cells were infected and analyzed by bright field microscopy 2 days after the infection, to directly examine if infected cells proliferate. As shown in Figure 7b, M059K cells did not propagate 2 days post-infection, indicating that the G1 population of M059K infected cells includes G1-arrested cells.

To study the functionality of the G1 checkpoint in AAV-infected M059K cells, protein analysis was performed. The G1 checkpoint is mainly dependent on two pathways: the p53/p21 and the p16/pRb, with the latter being important in p53-deficient cells [34]–[36]. M059K cells have a mutation in p53 and therefore a compromised p53/p21 pathway [37], which was confirmed by assaying for the levels of p21 after infection and showing that they are low (Figure 7c). To investigate if the p16/pRb pathway is functional, the levels of cyclin E were examined. Cyclin E expression is inhibited after binding of pRb to E2F, preventing cells from proceeding to S-phase [38]. Infected M059K cells showed a decrease in the levels of cyclin E, which would suggest that the cells are arrested at G1 through the p16/pRb pathway. This was further confirmed by assaying Cdc25A and showing that the levels of this protein also dropped 1 day after infection. On the other hand, U2OSp53DD cells do not have an operational p53/p21 pathway due to the expression of a defective p53 protein that acts in a dominant-negative way [30]. This was checked by assaying for p21 levels and showing that this pathway is indeed non-functional (Figure 7d). The p16/pRb pathway was then examined by investigating the levels of phosphorylated pRb. We found that this pathway is also compromised, as pRb was phosphorylated 1 day post-infection, indicating that the cells were not arrested at G1. This result is in line with a study showing that p16 expression is inhibited in U2OS cells [39]. The increase in the levels of Chk1 that we observed 1 day post-infection may explain the G2 arrest established in infected U2OSp53DD cells. These results thus confirm that M059K cells have a functional G1 checkpoint, while the U2OSp53DD cells do not and arrest in G2 after AAV infection.

To check that AAV infection of M059K cells is efficient, cells were infected with untreated wild type AAV and then stained for AAV capsid proteins (Figure 7e). The data show that the glioblastoma cells are as well-infected as the U2OSp53DD cells. Finally, to confirm that AAV induces a DNA damage response in M059K similar to that seen in U2OSp53DD cells, M059K cells were infected with AAV and analyzed for the presence of DNA damage response foci. Cells were stained for phospho-RPA32, a marker of the DNA damage response provoked by AAV-induced stalled replication forks [40]. As shown in Figure 7f, the infection induced formation of DNA repair foci in M059K cells, similar to those seen in U2OSp53DD cells. Taking our results together, we conclude firstly that, although we found no expression of apoptotic markers during mitotic catastrophe in U2OSp53DD cells, the assays used readily revealed apoptosis when it was indeed occurring. Secondly, the specific response of cancer cells to AAV infection is not only cell-type-dependent, but also can vary within a single cell population.

## Discussion

### Mitotic cell death by multipolar spindle-induced cell disruption

Mitotic catastrophe is a type of cell death that occurs in cells with defective checkpoints and may have several causes, one of which is centrosome overduplication. In the experiments we report here we have tackled the question of the relation between mitotic catastrophe and programmed cell death. To do this, we used a unique reagent - a UV-inactivated version of the virus AAV. This virus is particularly informative for this study because it can initiate a strong and clearly defined DNA damage signal, while not causing damage to the DNA of the host cell [27], [32]. It is the viral DNA itself that is sensed by the cell as a stalled replication fork, due to its inability to complete replication. The use of wild type or inactivated AAV is interesting as a potential tool against cancer, as it has been shown to kill p53-deficient cells and prevent tumorigenesis in mice [30].

Our results show that mitotic cell death is induced by AAV in p53-deficient U2OS cells and that this can take place in the absence of apoptosis. In these cells, AAV infection leads to prolonged mitosis, overduplication of centrosomes, multipolar spindles and formation of micronucleated cells. To investigate further the way the AAV-infected cells die, we looked for markers of caspase-dependent apoptosis and found that the infected U2OSp53DD cells die independently of caspase activity. The cells were further shown not to have fragmented chromatin or to show signs of caspase-independent apoptosis. The processes of autophagy and necroptosis were also found not to be activated. Together with time-lapse microscopy of infected cells, these data suggest that the infected p53-deficient osteosarcoma cells die in mitosis due to mechanical collapse rather than apoptosis. Although in this work we concentrated on the well-defined DNA damage signal due to AAV infection, we have also shown that doxorubicin can elicit a similar response. Note that the parental U2OS cells are not a good control for U2OSp53DD because, due to p14 (ARF) promoter methylation, they have a p53 pathway that is already partly defective [39].

**Figure 8:**
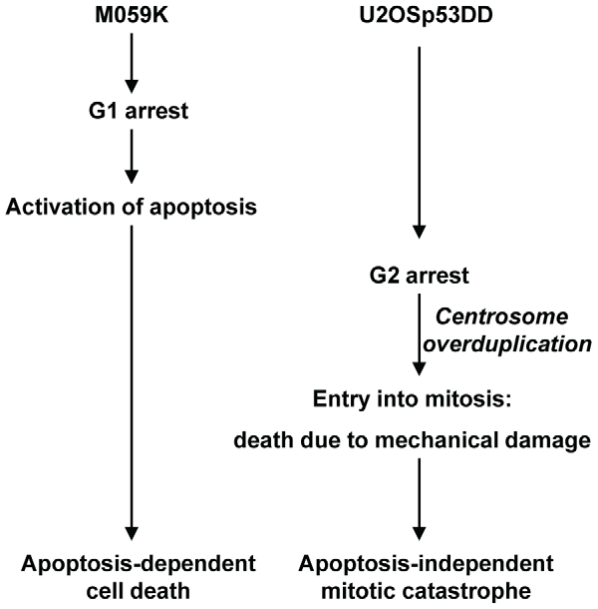
Hypothetical model explaining the difference between U2OSp53DD and M059K cells in deciding which cell death pathway to follow after AAV infection.

In contrast to U2OSp53DD cells, M059K glioblastoma cells that were infected with AAV responded differently. These cells arrested in the G1 phase of the cell cycle soon after infection and died 4 days post-infection. Death of these cells was caspase-dependent and was characterized by various hallmarks of apoptosis. M059K cells have a compromised p53/p21 pathway, due to the absence of functional p53 [37]. On the other hand, they appear to have a functional p16/pRb pathway, since cyclin E levels dropped 1 day after infection. Moreover, Cdc25A levels diminished 1 day post-infection. Given that the p16/pRb pathway is a p53-independent activator of G1-arrest [41]–[43], these results indicate that the glioblastoma cells activated their G1 checkpoint after AAV infection. It is therefore possible that activation of apoptosis in M059K glioblastoma cells is related to the G1 checkpoint activation and subsequent G1 arrest that is seen in these cells, so that abrogating this checkpoint would affect the frequency of apoptosis (Figure 8). G1 checkpoint-competent NIH3T3 cells also could undergo apoptosis, in line with studies showing G1-phase-dependent activation of apoptosis [44], [45]. Nevertheless, since U2OS and M059K are not isogenic, more work is needed to define with certainty the determinants of apoptosis.

The details of how AAV-triggered DNA damage signaling may lead to apoptosis remain to be clarified. A model for Saos-2 cells has recently been proposed [46]. These cells are deficient in both p53 and pRb, and die through caspase-dependent apoptosis rapidly after AAV infection, with no signs of mitotic catastrophe being reported. Saos-2 cells have been shown to be more sensitive to AAV infection when compared to U2OSp53DD cells, with death pathways being activated soon after infection [30]. We propose that complete absence of pRb in Saos-2 cells renders them susceptible to apoptosis, which is triggered immediately after infection. Indeed, there are several studies showing that the absence or degradation of pRb is linked to immediate apoptosis induction [47], [48]. We therefore attribute the different responses of Saos-2 and U2OSp53DD cells to the different pRb status of the two cell lines. To know whether the Saos-2 model applies by analogy to other cell types will need further testing.

U2OSp53DD cells were unable to activate apoptosis and died due to cellular collapse in mitosis. These cells are defective in the p53/p21 pathway, as well as in the p16/pRb pathway [39]. This was confirmed by assaying for the levels of p21 and phosphorylated pRb. U2OSp53DD cells are therefore unable to activate the G1 checkpoint and sustain a G1 arrest. Nevertheless, these cells were able to arrest in the G2 phase 1 day post-infection, mainly due to a transient increase in the levels of activated Chk1 that prevented them from entering mitosis [27], [49]. We have previously shown that Chk1 levels decrease in U2OSp53DD cells 4 days after infection, releasing them into catastrophic mitosis [28]. Since the majority of the infected cells display amplified centrosome numbers, we propose that the Chk1-mediated G2 arrest gives time to centrosomes to reduplicate, causing the multiplicity of spindle poles observed in infected U2OSp53DD cells [17], [50]. The absence of p21 activation, which is normally required for regulating centrosome amplification and preventing mitotic entry, supports this conclusion [24], [51]. The time-lapse microscopic images in Movies S1 and S2, see also [29], showing the contents of rounded-up AAV-infected mitotic cells being tugged repeatedly back and forth, together with the absence of any signs of programmed cell death, lead us to propose that these cells enter mitosis, where they die due to mechanical failure of proper chromosome segregation, caused by the abnormal number of spindle poles (Figure 8).

### Cell-dependent responses to AAV infection

Why is treatment with genotoxic agents often lethal for cancer cells? Here we show that different cell-types can respond quite differently to exactly the same damage signal, UV-inactivated AAV. The underlying cause of this variability is probably complex, depending on the specific set of checkpoint mutations present. One criterion suggested by our findings is a functional G1 checkpoint. In this model, after a DNA damage signal induced by AAV in the presence of a competent G1 checkpoint and the consequent G1 arrest, certain cells are able to initiate a caspase-dependent apoptotic pathway and undergo cell suicide. Absence of the G1 checkpoint leads to escape from apoptosis. Nevertheless, those cells still die, but only after a mechanical catastrophe that is due to an unscheduled entry into abnormal mitosis. U2OS cells without the dominant negative p53, on the other hand, are more resistant to AAV infection, and show a strong cell cycle arrest, which is overcome after about 5 days [30].

Alternatively, it may be that a cell's sensitivity or indifference to apoptotic signaling underlies the variation seen in response to AAV infection. Our FACS analyses of cells treated with the pan-caspase inhibitor suggested that even within a single cell population the response to AAV infection varies. This is confirmed by quantitative live imaging showing that while over 80% of the infected U2OSp53DD cells die in a prolonged aberrant mitosis, the remaining cells respond differently and rapidly undergo apoptosis [29]. These findings are consistent with a model in which non-genetic determinants of cell fate, such as the balance between signals promoting apoptosis or continued movement through the cell cycle, can vary within individual cells with time.

The principal outcome of our study is that mitotic catastrophe can occur independently of apoptosis. Mitotic cell death in p53-null cells occurred predominantly as a result of mechanical disruption induced by multipolar mitotic spindles, and not as a consequence of a suicide signal. Although chromosomal instability has been considered to be tumorigenic, it is now becoming clear that, paradoxically, high rates of aneuploidy may cause tumor suppression and cell death [52]. Our results suggest that AAV or UV-inactivated AAV can be used as an inducer of DNA damage signaling to target p53-deficient cells and trigger death after entry of cells with overamplified centrosomes into mitosis. Since this does not involve apoptosis, cell death triggered by AAV, or by drugs that interfere with cell cycle control and mitosis in a similar way, may still target cells that have lost apoptotic signaling pathways.

## Materials and Methods

### Cell lines and chemicals

M059K and NIH3T3 cells (American Type Culture Collection) were maintained in Dulbecco's modified Eagle's medium (DMEM) supplemented with 10% fetal bovine serum (FBS), penicillin/streptomycin and ciprofloxacin (Ciproxin; Bayer, Leverkusen, Germany). U2OSp53DD cells, a kind gift from Dr. K. Raj who produced them [30], were maintained in the above medium supplemented with 1.5 µg/ml of puromycin. Chemicals used are the following: ActinomycinD (1 µg/ml), chloroquine (50 µM), DNaseI (0.5 u/µl), doxorubicin (50 nM), necrostatin-1 (80 µM) nocodazole (0.1 µg/ml), staurosporin (200 nM), TNFα (50 ng/ml) and zVAD-fmk (20 µM). All chemicals were from Sigma-Aldrich (Dorset, UK), except DNaseI (Roche, Basel, Switzerland), necrostatin-1 (Alexis, Lausen, Switzerland) and TNFα (Cell Sciences, Canton, MA, USA).

### Virus production and infections

Production of AAV was done in HeLa cells and has been previously described [53]. All experiments were performed using UV-inactivated AAV2, unless stated otherwise. UV-treatment of AAV and subsequent infection have been described before [32].

### Propidium Iodide staining and FACS analysis

The PI-staining protocol has been previously described [40]. Flow cytometry was performed using a FACScan Becton-Dickinson (San Jose, CA, USA) or a CyAn DAKO (Glostrup, Denmark) flow cytometer.

### Immunofluorescence staining and microscopy

The procedure for IF-staining and microscopy analysis has been extensively described [32]. The primary antibodies used were the following: anti-AAV-capsid (Progen, Heidelberg, Germany), anti-AIF (Cell Signaling, Danvers, MA, USA), anti-α-tubulin (Abcam, Cambridge, UK), anti-cleaved-caspase3 (Cell Signaling), anti-cleaved-PARP1 (Cell Signaling), anti-LC3B (Cell Signaling) and anti-phospho-RPA32-S4/S8 (Bethyl, Montgomery, TX, USA). The secondary antibodies used were Alexafluor-488 (Molecular probes, Eugene, OR, USA) and Cy3 (Jackson Immunoresearch, West Grove, PA, USA) IgG conjugates. The annexin-V-Fluos staining kit (11 858 777 001) was obtained from Roche Diagnostics GmbH and used following the manufacturer's instructions. Images were obtained using the Zeiss Axioplan microscope and were acquired with an AxioCam MRm Zeiss camera, using the Axiovision 4.5 software. The 63× objective was used, unless otherwise stated. For live imaging, the Zeiss Time-laps Axiovert 100 microscope was used.

### Western blotting

The detailed protocol has been described before [32]. The primary antibodies used were the following: anti-α-tubulin (Abcam), anti-Bax (Santa Cruz), anti-Cdc25A (Santa Cruz), anti-Chk1 (Abcam), anti-cyclin E (Santa Cruz), anti-mcm3 (Abcam), anti-p21 (Santa Cruz), anti-cleaved-PARP1 (Cell Signaling) and anti-pRb (BD Pharmigen, San Diego, CA, USA). Horseradish peroxidase-conjugated IgGs were used as secondary antibodies (Jackson Immunoresearch).

### Tunel assay

The fluorescein FragEL DNA fragmentation detection kit (Calbiochem, San Diego, CA, USA) was used and the protocol described by the manufacturer was followed.

## Supporting Information

Figure S1 **Inhibition of microtubule polymerization prevents cell death in mitosis.** (a) U2OSp53DD cells were infected with UV-AAV and treated with nocodazole (Noco) 1 day before IF analysis, to prevent microtubule polymerization. Samples were analyzed 4 days after infection for micronucleated cells by DAPI staining. α-tubulin was used as a control for the effectiveness of the nocodazole treatment. Indeed, α-tubulin did not stain polymerized microtubules in the AAV-infected/nocodazole-treated sample. Furthermore, treatment with nocodazole alone resulted in a large number of cells arrested in prometaphase, as seen by their condensed chromatin and the staining of condensed unpolymerized α-tubulin. Images were acquired using the 10× objective. Arrows indicate micronucleated cells. Bar: 230 µm. (b) The experiment described in (a) was replicated and the average percentage of micronucleated cells was calculated in infected cells with or without nocodazole treatment. Nocodazole treatment itself resulted in a small but significant number of micronucleated cells. Error bars represent standard deviations.
(TIF)

Movie S1 **Time-lapse video of AAV-infected cells from the experiment described in** **Figure 1d** **.**
(AVI)

Movie S2 **Time-lapse video of untreated cells from the experiment described in** **Figure 1d** **.**
(AVI)
