# Supplementary figures and images for: Mitotic Catastrophe Occurs in the Absence of Apoptosis in p53-Null Cells with a Defective G1 Checkpoint
Source: PLoS One. 2011 Aug 10;6(8):e22946. doi: 10.1371/journal.pone.0022946 (PMC3154265; doi:10.1371/journal.pone.0022946)

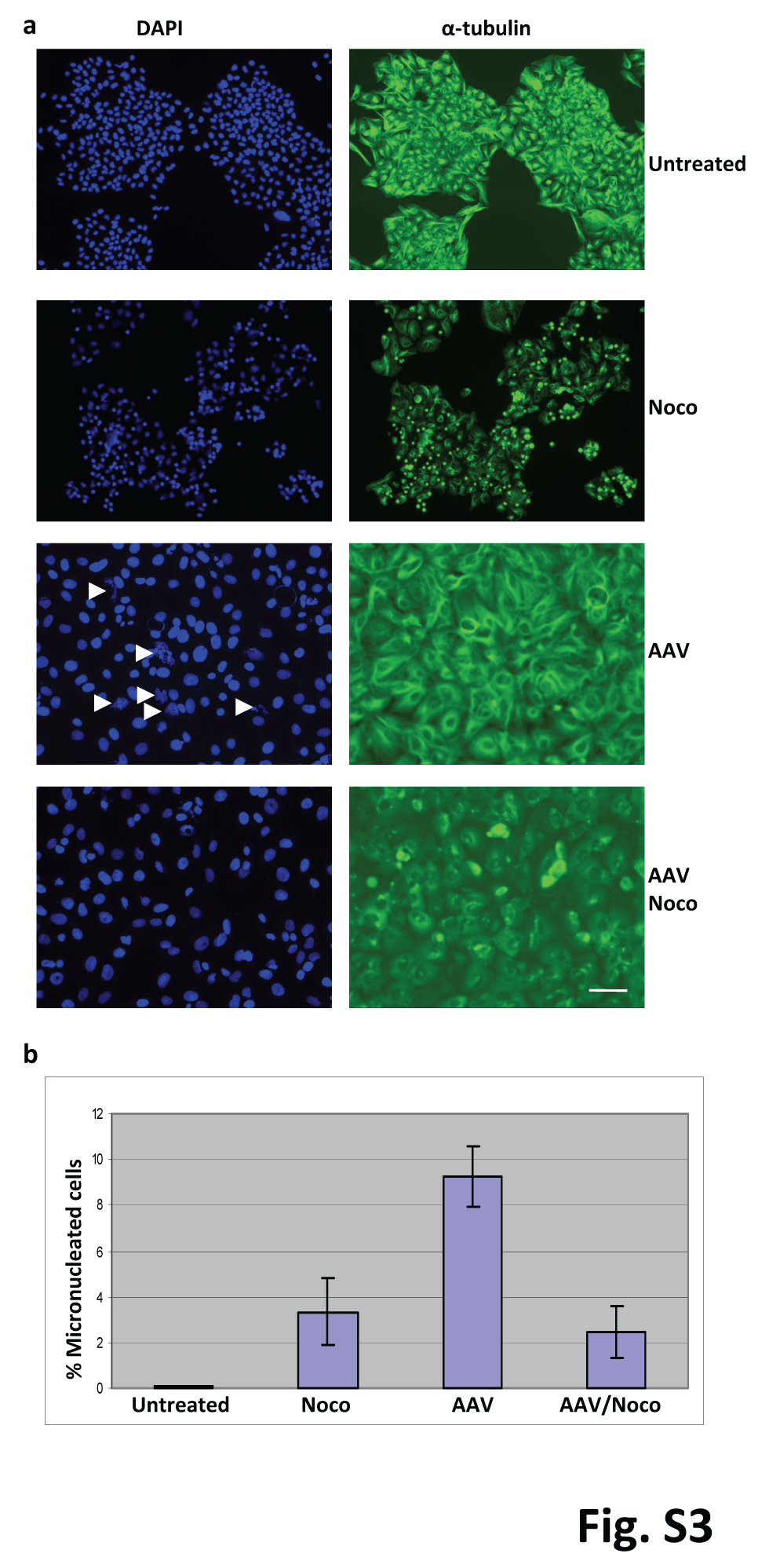

Supplement: Figure S1 — Inhibition of microtubule polymerization prevents cell death in mitosis. (a) U2OSp53DD cells were infected with UV-AAV and treated with nocodazole (Noco) 1 day before IF analysis, to prevent microtubule polymerization. Samples were analyzed 4 days after infection for micronucleated cells by DAPI staining. α-tubulin was used as a control for the effectiveness of the nocodazole treatment. Indeed, α-tubulin did not stain polymerized microtubules in the AAV-infected/nocodazole-treated sample. Furthermore, treatment with nocodazole alone resulted in a large number of cells arrested in prometaphase, as seen by their condensed chromatin and the staining of condensed unpolymerized α-tubulin. Images were acquired using the 10× objective. Arrows indicate micronucleated cells. Bar: 230 µm. (b) The experiment described in (a) was replicated and the average percentage of micronucleated cells was calculated in infected cells with or without nocodazole treatment. Nocodazole treatment itself resulted in a small but significant number of micronucleated cells. Error bars represent standard deviations. (TIF) [file pone.0022946.s001.tif]
